# The Effect of Lift Crates on Piglet Survival Rate and Sow Stress Level during Farrowing

**DOI:** 10.3390/ani12060745

**Published:** 2022-03-16

**Authors:** Annamaria Costa, Cecilia Salvagnini, Eleonora Buoio, Fabio Palmeri, Andrea Salvagnini, Silvia Michela Mazzola

**Affiliations:** 1 Department of Veterinary Medicine and Animal Sciences, Università degli Studi di Milano, Via dell’Università 6, 26900 Lodi, Italy; annamaria.costa@unimi.it (A.C.); eleonora.buoio@unimi.it (E.B.); 2 Department of Biotechnology, University of Verona, Strada Le Grazie 15, 37134 Verona, Italy; csalvagnini97@gmail.com; 3 Forester, Verona, Italy; fabio.palmeri@tecnovia.it; 4 Agronomist, Verona, Italy; asalvagnini@studioterra.it

**Keywords:** farrowing crate, lift crate, piglet, crushing rate, sow welfare, chronic stress, hair cortisol concentration

## Abstract

**Simple Summary:**

In intensive farms, the mortality of newborn piglets during farrowing is a critical aspect that represents a significant cause of economic loss in pig production, and the deaths, representing 5–25% of newborn piglets, depend mainly on starvation and crushing. In the present study, we aimed to determine the effect of lift farrowing crates on piglet mortality by crushing and on sow welfare. Eighty-four sows of the same genetics were observed over three sessions for one year. Twenty-eight sows per session were randomly assigned to a room equipped with conventional crates or lift crates. No primiparous sows were considered in the study to avoid sows unexperienced with the dynamics of lift crates. The farm’s veterinarian assessed the number of crushed piglets within 48 h, 72 h, and at weaning. Hair cortisol concentration (HCC) was measured at the entry and the exit from farrowing to evaluate stress level variation. Feet diseases and backfat thickness were evaluated to assess sows’ potential diseases induced by lift crates and metabolic problems. The results show that the number of crushed piglets per sow was higher in the conventional crate rooms than the lift crate ones. Mean values of HCC variation in sows during farrowing were significantly different in the two housing systems and higher for the lift crate-housed sows. No significant differences were detected for backfat variation and feet disease scoring. In conclusion, sows housed in the lift crates evidenced an increase in hair cortisol values during farrowing, probably reflecting a higher stress status induced by the lift crate.

**Abstract:**

In the present study, we aimed to determine the effect of lift farrowing crates on piglet mortality by crushing and on sow welfare. Eighty-four sows were considered in the one-year experiment in three monitoring sessions. In each session, 14 sows were housed in a room with conventional crates (CC), and 14 sows were lodged in a room equipped with lift crates (LC). The sows, of the same genetics, with parity ranging from 2 to 9, were randomly distributed in CC and LC rooms. No primiparous sows were considered in the study to avoid sows unexperienced with the dynamics of lift crates. The numbers of crushed piglets, assessed by the farm’s veterinarian, within 48 h, 72 h, and at weaning (28th day), were recorded. Hair cortisol concentration (HCC) was measured upon entry and exit from farrowing to evaluate stress level variation. Feet diseases and backfat thickness were evaluated to assess sows’ potential diseases induced by lift crates and metabolic problems. The results show that the number of crushed piglets per sow was higher in the CC rooms than in the LC rooms in the first two days after delivery (0.39 vs. 0.15, *p* < 0.05) and up to weaning (0.50 vs. 0.37; *p* < 0.05). Mean values of HCC variation in sows during farrowing were significantly different in the two housing systems and higher for the LC sows (0.53 pg/mg vs. 0.22 pg/mg; *p* < 0.05). No significant differences were detected for backfat variation and feet disease scoring between LC and CC sows. In conclusion, LC sows evidenced an increase in hair cortisol values during farrowing, probably caused by a higher stress status induced by the lift crate, along with the benefit of the higher survival rate of piglets before weaning.

## 1. Introduction

The mortality of newborn piglets during farrowing is a critical aspect that represents a significant cause of economic loss in pig production [1].

Many parameters determine piglets’ mortality rate before weaning, such as genetics, management, environment, housing system design, nutritional status, infectious diseases, litter size, and the maternal attitude of the mother towards the newborn piglets [2,3]. It has been observed that in intensive farms, 5–25% of newborn piglets die before weaning, mainly due to starvation (up to 43%) and crushing (20–40%); these causes could explain 50–80% of piglet deaths before weaning [4]. Moreover, an inadequate microclimate [2,5] and poor maternal attitude of the sow (5–15%) can worsen piglets’ survival rate. Crushing by the mother sow is usually related to an uncomfortable design of the pen, which can play an essential role in piglet survival at weaning [6,7].

The first days of farrowing are the most critical for piglets: in the first 72 h, the mother can crush some newborns during her rolling movements [8,9]. The number of crushed piglets is related to the sudden movements of the sows, combined with the significant size difference between mother and offspring—these factors may cause suffocation and serious injuries, with consequent death of the newborn piglet [2]. The term “crushing” implies that death is due to trauma to the piglet’s body by the sow; however, many piglets are killed by suffocation when trapped under the body of the sow for a prolonged time, and not as a direct result of traumatic injury [10].

Another risk factor implied in crushing is sows’ lameness, a complex of wounds and lesions that may also lead to sows’ early culling [11,12], which increases economic losses for pig farms.

Starting in the 1950s, the introduction of farrowing crates confining the sow represented the most secure way to protect piglets from crushing, reducing their mortality rate [5,13] and optimizing the available space in the farrowing room [4]. However, the crate also limits the sow’s movements and, unfortunately, her ability to exhibit maternal behavioral patterns [6].

Nowadays, different stall types are used in pig farms to prevent the death of newborn piglets by crushing and to improve sows’ welfare status in the farrowing period. 

In the past two decades, the concept of farrowing crates raised societal concerns about animal welfare, and the restricted area housing the sow in the crate is seen as detrimental to her welfare by the public [5,14]. The European Parliament, in June 2021, after the results obtained by the petition “End of the Cage Age” according to P9_TA (2021-2633, https://oeil.secure.europarl.europa.eu/oeil/popups/printficheglobal.pdf?id=725281&l=en, accessed on 30 November 2021) asked the Commission to propose legislative instruments on fair and sustainable farming, and in particular, to propose a revision of Directive 98/58/EC with the objective of phasing out the use of cages in EU animal farming, assessing a possible phasing-out by 2027, starting from the recognition of available literature on pig legislation and welfare, structural environment, and stall design.

Following these societal concerns, innovative solutions were conceived to improve animal welfare conditions and maintain high productivity in intensive farms, such as the free farrowing system, aimed at improving sows’ physiological and behavioral conditions [15]. Nevertheless, the higher crushing rate of piglets compared to the number of newborns crushed in the farrowing crates limits the adoption of non-crated farrowing systems in piggeries [16].

The European Food Safety Authority in 2007 expressed caution towards the adoption of farrowing pens given the increased risk of higher mortality by crushing in loose housing systems [15]. Moreover, the greater space allowance can induce a higher mortality rate before weaning, even in the second farrowing phase, from the 15th day on [17].

Recently, a new farrowing crate was produced, the lift crate, which is essentially an enclosed conventional farrowing crate that can lift the sow from the ground depending on her posture. A bar equipped with a sensor activates the lift when the sow changes posture, touching the bar with the dorsal region while standing to feed in the trough. When the sow returns to lay on the floor, the lack of sow/bar contact brings the sow back to the creep level, avoiding the direct contact of the sow with piglets during these movements.

These controversial aspects in pig farming need the objective assessment of the welfare status of sows in crates, using welfare indicators to evaluate stress levels during farrowing, without emotive considerations.

In the past, the term “stress” was only indirectly used to measure the level of animal welfare, traditionally defined as the combination of the five freedoms [18]. In recent years, welfare assessment moved from detecting temporary stress following critical situations to evaluating chronic stress for measuring welfare in the different stages of animals’ productive life [19].

Usually, the most used indicator to assess stress levels is cortisol. The adrenal cortex produces this glucocorticoid under the stimulation of the adrenocorticotropic hormone (ACTH), and it is released in body fluids in most mammals [20] as a response to stressors by the activated hypothalamic–pituitary–adrenal axis [21].

Body fluid cortisol can be used as a biomarker to investigate acute stress [22]; it can be detected in the blood, saliva, milk, urine, and feces [23]. Cortisol and glucocorticoids in general reach the maximum peak within 30 min after the stressor event, with the negative aspect of extreme variability among individuals [18].

On the contrary, the use of hair cortisol to assess chronic stress in animals, pioneered by Koren et al. in 2002 [24], represents a reliable tool to obtain information on long-term HPA axis activity for weeks prior to the sampling day [25] and to determine the long-term or chronic stress in a non-invasive way [26,27], including in pigs [28].

For the reasons mentioned above, we aimed to evaluate the effects of innovative lift crates compared to conventional crates on the piglet mortality rate by crushing and on sows’ welfare during farrowing through hair cortisol titration. 

## 2. Materials and Methods 

### 2.1. Ethical Statement

The farm at which this experiment was conducted complied with Statutory Instrument number 311 of 2010 European Communities (Welfare of Farmed Animals) Regulations 2000. Ethical review and approval were waived for this study, as the guidelines of the Italian law for the care and use of animals (D.L. 04/3/2014 n. 26) and the EU directive (2010/63/EU) indicate that all methods and procedures used in this study fall within those excluded from the need for approval, as no invasive measures were used.

### 2.2. Animals, Location, Housing, and Management

The experimental study was conducted in a pig farm located in Santa Maria di Zevio (VR), northern Italy, from November 2018 to August 2019 (Table 1). 

Eighty-four sows (Landrace × Large White × Duroc, a typical cross for Parma ham production) were involved in the trial, organized in three monitoring sessions. During the three monitoring sessions, 14 sows were housed in a room with conventional crates (CC), and 14 sows were lodged in a room equipped with lift crates (LC). The sows, with parity ranging from 2 to 9, were randomly distributed in CC and LC rooms by order of birth and genetics. No primiparous sows were considered in the study to avoid sows unexperienced with the dynamics of lift crates; all the sows involved in the study were housed at least once before in a lift crate. 

The production cycle in the farrowing rooms lasted five weeks. Sows were moved into the farrowing rooms one week before delivery and left the room at piglets’ weaning four weeks later. A sanitary vacuum period of 3 days was applied for washing and disinfection.

The CC and LC rooms were similar in design, structural organization, feeding formulation, feeding system (Spotmix feeding system, Schauer, Prambachkirchen, Austria), and management. The ventilation system, equipped with inlets at the animal level, was managed by FRCA control units (FANCOME, Panningen, The Netherlands) based on a free-running impeller (FANCOM EasyFlow, Panningen, The Netherlands) in each farrowing room, for continuous and real-time monitoring of the ventilation rate to maintain the required temperature. Both solutions were equipped with a heating system, and each crate had a heated nest (infrared lamps, 150 W) for piglets in the creep area.

#### 2.2.1. Lift Crates

**Figure 1:**
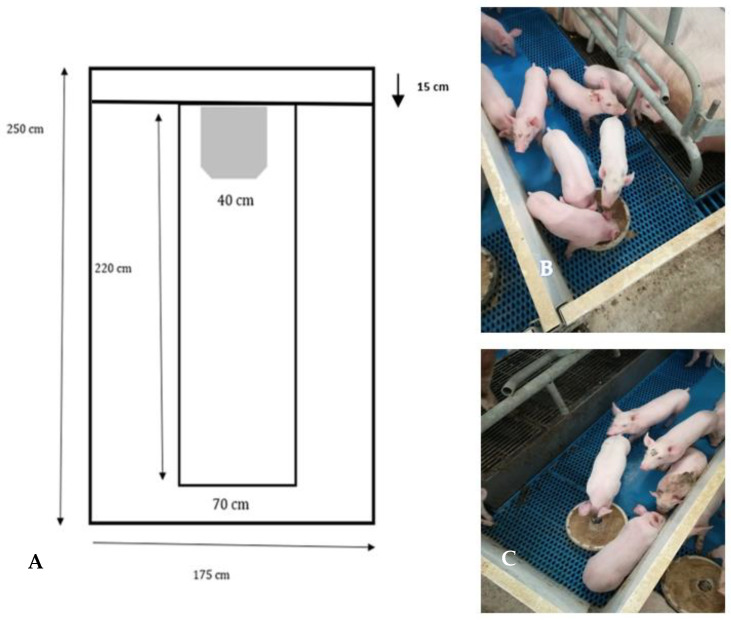
(**A**–**C**) Design and photos of the lift farrowing crate (LC).

The lift farrowing crates (Nooyen Balance frame, Nooyen pig flooring, Deurne, The Netherlands) were equipped with self-propelled floors, which, thanks to a sensor detecting the animal’s standing position, raises the floor of the sow 28 cm higher than the creep area housing the piglets, through an air hydraulic system. When the sow lies down in the decubitus position, ready to feed piglets, the sensor lowers the floor to the piglet level. The details of the LC crate are presented in Figure 1A–C. 

The LC pens cover a total area of 41,125 cm^2^, with the creep area being 25,725 cm^2^ wide and the pen area for the sow being 14,700 cm^2^ wide (Figure 1A). In front of the sow, there is an inlet for fresh air, 15 cm in depth. In Figure 1B, the sow is lying down at the creep area level. In this case, the sensor, positioned in the side containment bars, is not in touch with the sow. In Figure 1C, the sow is standing, the bar containing the sensor is touching her back, and the floor of the crate is lifted 28 cm above the creep area.

#### 2.2.2. Conventional Crates

**Figure 2:**
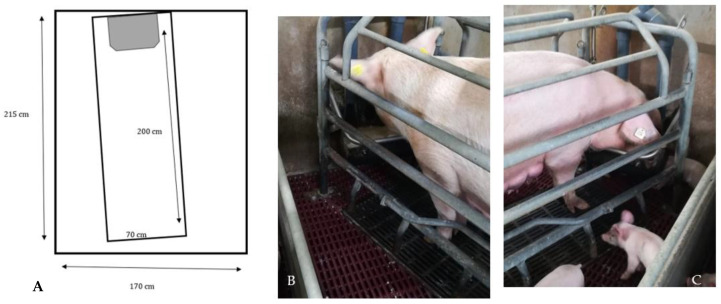
(**A**–**C**) Design and photos of the conventional farrowing crate (CC).

The conventional crate pens cover a total area of 36,550 cm^2^, with a creep area 22,550 cm^2^ wide, and a pen area for the sow 1400 cm^2^ wide (see Figure 2A). 

The CC is equipped with containment bars to prevent the sow from climbing, as well as inclined bars, arranged on the lower beam, to limit the sow’s movements in order to not cause injuries to newborn piglets (Figure 2B,C).

The farrowing pen has a cast-iron slatted floor, one nipple for the sow and one for the piglets. The nest is positioned in the front area, and it is equipped with an IR lamp to keep piglets warm during resting time.

#### 2.2.3. Climatic Conditions in the Rooms 

Climatic conditions in the rooms (temperature and ventilation rate) were monitored with one hour of frequency, downloading data from the Fancom ventilation system. Temperature and relative humidity were monitored using a datalogger able to monitor and store temperature and relative humidity (LASCAR, RS Components S.r.l., Sesto San Giovanni, Milan, Italy) with 15 min of frequency.

### 2.3. Piglet Performance 

The number of piglets born alive, born dead, mummified, and crushed from delivery to 72 h of age and at day 28 (weaning) were detected and recorded by the farm’s veterinarian, performing necropsies on dead and stillborn piglets. Piglets were identified as “crushed” when they showed signs of traumatic injuries, bruises, or imprints of the floor. 

### 2.4. Hair Samples for Hair Cortisol Concentration Measurements (HCC)

**Figure 3:**
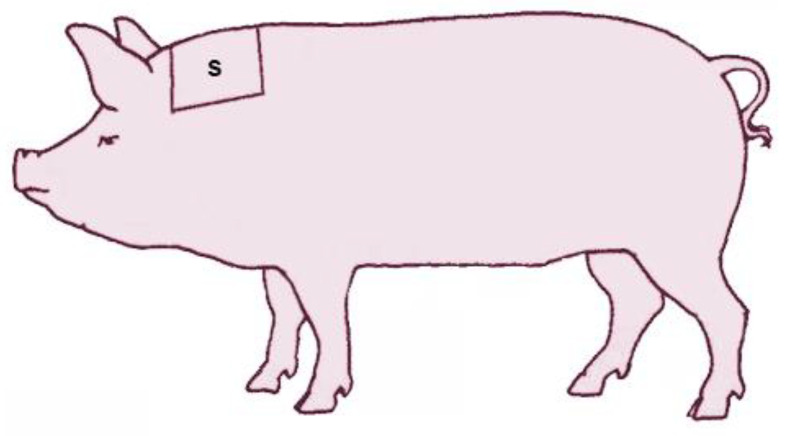
Localization of the shaving area between neck and shoulder in sows.

A bilateral symmetric area of 20 × 20 cm was shaved in the transition area between the neck and shoulder blades (Figure 3), as close as possible to the skin, according to Wiechers et al. [18]. Two sampling timepoints were chosen, upon entry and exit from the farrowing room.

Hair samples were stored in polypropylene tubes (Falcon, Fisher Scientific, Rodano, Milan, Italy) at room temperature until analysis. Extraction of hair cortisol was performed following the procedure described by Burnett et al. [29,30]. A 100 mg hair sample from each sow was washed twice with double-distilled water and allowed to dry overnight. Hair samples were then washed with isopropanol for 3 min and allowed to dry completely overnight. The washed and dried hair was powdered in a ball mill (Retsch MM400, Retsch-Allee, 1-5 42,781 Haan, D) and 40 mg of clean and dried hair was weighed in vials; then, 2 mL of 99.9% methanol (Sigma-Aldrich, Milano, Italy) was added. The vials were tightly capped and sonicated for 30 min (Branson 2510, Branson Ultrasonic Corp, Danbury, CT, USA). The samples were then incubated overnight at 100 rpm and 50 °C to extract the steroids, and then 1.5 mL of the original volume of methanol was pipetted into a 2.5-microcentrifuge tube and evaporated at 45 °C under a stream of ultrapure nitrogen gas. The samples were reconstituted in 200 μL of phosphate-buffered saline (Merk Millipore, Milano, Italy). Hair cortisol was analyzed using a commercially available assay kit designed to accurately measure cortisol levels in a variety of sample matrices (Enzo Life Sciences, Farmingdale, New York, NY, USA). Samples were aliquoted into wells in duplicates (100 µL), and absorbance was measured in a microplate reader (Multiskan EX, LabSystem, Thermo Fisher Scientific, Milan, Italy), using a wavelength of 405 nm. The samples’ pg/mL cortisol concentrations were calculated from the standard linear curves. The laboratory researcher was blinded to the hypotheses and conditions.

### 2.5. Measurement of Sows’ Backfat Thickness 

Backfat thickness variation in sows was measured to investigate different potential responses, not depending on the used crate type, but from a different energy income by the animals. Sows’ backfat thickness was measured by an ultrasound Renco Lean-Meater (Renco Corporation, distributed by Schippers, Azzano Lombardo, Italy) at two time points. The instrument uses pulsed ultrasound to measure the total backfat depth of mammals with 1, 2, or 3 backfat layers. Total measurement range and accuracy, including skin, is 4–35 mm, ±1 mm. Measurements were conducted twice during the farrowing cycle: five days before expected delivery or entering the farrowing room, and at weaning (after 28 days of lactation). The measurements were performed in the back area at point P2 of the rib, 6–7 cm lateral to the dorsal midline. Before the measurement, the selected area was shaved, designated with a color marker, and greased with paraffin oil. The average of three successive measurements was taken.

### 2.6. Locomotion and Feet Lesions Detection on Sows 

Sows were scored for locomotion and front and rear feet lesions at the entrance and exit from farrowing to detect potential feet diseases induced by the LC crates. The scoring was performed by the veterinarian of the farm, observing sows’ locomotion as they walked from the gestation area to the farrowing room, covering a distance of around 50 m. The veterinarian evaluated feet lesions upon sows’ entry into the crates, waiting for sows to rest on the floor. At the end of farrowing, the farm veterinarian evaluated the feet lesions before the sows’ exit from the crates, and observed the sows walking around 70 m to the heat detection/insemination room to detect problems in locomotion. The veterinarian classified feet disease by lifting and observing the feet. Lameness was detected using a system developed by the Feet First team [31], assigning a score from 0 to 3 to assess the severity of locomotion problems and lameness in sow. The system effectively allows early diagnosis of foot disorders and lesions and permits monitoring the prevalence of lameness in sow farms.

#### 2.6.1. Locomotion Scoring

Locomotion scoring was assessed according to a 3-level scale based on the observation of sows standing and walking:

0: Sow moves easily with little inducement and appears comfortable on all her feet.

1: She moves relatively easily, but visible signs of lameness are evident in at least one leg, with a reluctance to bear weight on that leg, but still moves easily from site to site in the barn.

2: Lameness is involved in one or more limbs. The sow exhibits compensatory behaviors, such as dipping the head or arching the back.

3: There is an evident reluctance to walk and bear weight on one or more legs, and the movements from place to place on the farm appear difficult.

#### 2.6.2. Feet Lesion Scoring

The lesion scoring was assessed considering the seven more common claw lesions, based on the type of lesion and its severity level (1 = mild, 2 = moderate, 3 = severe), as described in Table 2.

Other parameters detected were sows’ food intake, recorded as the difference between the amount of feed released by the Spotmix system and the residual feed in the trough at the end of the day, and reproductive problems in sows lodged in the experimental rooms after the monitored cycles.

### 2.7. Statistical Analysis

Data were tested for normality and submitted to variance analysis (Proc GLM for repeated statement of the SAS 9.4 statistical package, 2019) to assess the effect of the lift farrowing crates on the number of crushed piglets and sow stress levels, measured by hair cortisol concentration, feet disease, and backfat thickness variation after farrowing, in comparison with conventional crates.

Normal distribution of data was checked using histograms, and data not normally distributed were log-transformed to perform variance analysis. 

Sow and batch were considered as random effects.

Housing system (LC vs. CC), parity, and the interaction housing system x parity were considered as effects to be tested for crushing rate and cortisol variation as sows’ stress indicator, considering backfat thickness and feet diseases variation during farrowing. 

For each statistical analysis, the minimum significance level was declared at *p* < 0.05.

## 3. Results

### 3.1. Environmental Parameters in the Farrowing Rooms

Temperature and ventilation rate, set up by the ventilation system (see Table 3), were similar in the two types of farrowing rooms, as was expected. Relative humidity was in the range of 60–80%, as recommended for the compartment.

### 3.2. Piglets: Production Performance and Crushing Rate

All litters were cross-fostered and standardized to 14 pigs within 24 h after parturition, and all the sows delivered in 2 days. Cross-fostering was necessary to replace crushed or dead piglets and to standardize litters. No significant differences were reported for crushing rate according to parity; for this reason, the effect parity was deleted from the variance analysis. Table 4 shows the number of piglets born alive, born dead, mummified, and crushed within 3 days of delivery to day 28, the end of the farrowing cycle. The average number of piglets born alive in the three observation cycles in the two rooms is similar; no statistical differences were found for the number of stillborn piglets. The number of crushed piglets per sow was higher in the CC rooms, in comparison with the LC ones during the first three days after delivery (0.44 vs. 0.15, *p* < 0.05) and in total, up to weaning (0.48 vs. 0.37; *p* < 0.05).

### 3.3. Animal Welfare Parameters: Hair Cortisol (HCC), Back Fat, Locomotion, and Feet Lesion Scores

#### 3.3.1. Hair Cortisol

Table 5 shows the mean values of cortisol measured in sow hair upon entry into the farrowing crate and in hair grown during farrowing, shaved upon exit from the farrowing crate. Hair cortisol was significantly higher in LC sows than CC sows (0.53 Pg/mL vs. 0.22 Pg/mL; *p* < 0.05).

#### 3.3.2. Back Fat Measurements 

Table 6 shows the mean backfat thickness values measured at P2 on the sows during the three monitoring sessions. In the two groups of sows, the mean difference in backfat thickness of sows entering/leaving the farrowing room was not significantly different (2.34 mm for LC and 2.16 mm for CC sows).

#### 3.3.3. Lameness: Locomotion and Feet Lesion Scoring on Sows

Table 7 shows the mean values of locomotion and feet disease scoring.

Since data were not normally distributed, they were log-transformed to perform variance analysis.

The mean locomotion scoring values, ranging from 0 (no pathologies) to 3 (severe, the sow refuses to move because of pain), did not show differences in LC (mean score of 0.32 upon entry and 0.20 upon exit) vs. CC (mean score of 0.17 upon entry and 0.13 upon exit). 

Feet disease scoring data showed that only dew claws had higher values in LC sows, but data were not significantly different from CC sows.

No significant differences were detected between the data collected in the two crate types.

## 4. Discussion

In the present study, we aimed to determine if lift crates driven by postural changes in the sow can prevent piglet crushing and evaluate sow stress levels during farrowing in this housing system, using hair cortisol as a chronic stress indicator.

The study showed promising results in preventing piglet crushing when the mother is housed in the lift crate, compared to when the sow is housed in the conventional farrowing crate (0.44 vs. 0.15, *p* < 0.05) within 72 h after delivery, with a total crushed piglets per sow of 0.48 vs. 0.37, *p* < 0.05. 

The lift crate is an innovative crate, mainly adopted by Canadian farmers and on some farms in northern Italy. To our knowledge, only two references are available for lift crates, studies performed by Obermier et al. [32] and Mazzoni et al. [33]. Obermeier et al. [32] found that lift crates can significantly reduce crushing deaths by 55% when compared to conventional crates (0.58 vs. 0.32 piglets). Mazzoni et al. [33] reported 0.54 piglets crushed in lift crates vs. 5.46 piglets crushed in conventional crates. Both experimental studies report the efficiency of preventing death by crushing in this housing system. This efficiency depends mainly on the fact that the piglets reared in conventional farrowing crates are usually crushed due to the difficulty of walking for the newborns, especially in the first days of life, combined with their considerable size disproportion compared to the sow. Crushing by the sow is a predominant cause of death in crates and pens [34]: sows’ movements and rolling behavior, dependent on farrowing crate design, environmental, health, and management parameters, may be responsible for 18–36% [35] or up to 65–75% of the crushing rate, as reported by Weary et al. [10]. Sows’ risk movements could be prevented by adopting alternative farrowing systems, since the crushing rate still represents a significant factor in mortality before weaning in piggeries [1,3].

However, it should be emphasized that these observations about the efficiency of limiting the crushing rate in the crate housing system must be considered together with the welfare and health status of the sow during farrowing.

Today, no information is available about the effects of lift crates on the stress levels of farrowing sows in this dynamic housing stall. 

In the present study, hair cortisol was chosen as the first parameter to investigate the stress level experienced by sows lodged in the two different crates during the whole farrowing period. 

The levels of plasma cortisol typically rise during farrowing, and do not depend on the housing system [36]. However, higher cortisol levels were detected in crated sows compared to loose-housed sows during this time [36]. LC sows presented higher hair cortisol variations during the farrowing phase in the present study. Hair for cortisol extraction was shaved on the sows’ neck before their entry into the farrowing room and at the end of farrowing or the piglets’ weaning time (Shaving/re-shaving method). Collecting hair from the neck zone avoids sampling hair dirty with manure, which contains cortisol itself [18]. Casal et al. [37] found higher hair cortisol concentrations in the dorso-lumbar region of pigs compared to the cranio-dorsal region, probably due to the higher level of soiling with feces. This study’s results are consistent with those obtained by Wiechers et al. [18], who collected hair from the same body region of the sow, with a mean HCC of 1.99 ± 1.23 pg/mg for all analyzed samples. Bacci et al. [38] collected hair samples from different body sites, with different results in terms of cortisol concentrations.

Most earlier studies dealing with stress levels of crated and loose-housed sows in farrowing systems measured cortisol from saliva and blood. Lawrence et al. [36] implanted sows with jugular catheters approximately 10–14 days before the expected date of parturition (EPD) and found a significant rise in blood cortisol in sows lodged in conventional crates compared to sows housed in pens allowing freedom of movement with bedding; the difference was probably due to the close confinement inhibiting the expression of maternal behavior that induced stress in sow.

Biensen et al. [39] quantified cortisol in blood collected from surgically implanted vena cava cannulas in pre-parturient sows. The authors found a high variability of cortisol for sows in both pens and crates during the week before farrowing and during lactation; cortisol concentrations were significantly higher for sows in pens compared to sows in crates, probably due to different environmental conditions.

Goumon et al. [40] found that salivary cortisol concentrations in sows temporarily (up to day 3 post-partum) and conventionally crated to weaning were not affected by the different housing system.

In general, the literature shows a certain variability in cortisol concentrations measured in differently housed sows around farrowing.

In our study, backfat thickness, which can be considered an indicator of energy assumption and metabolism, was not affected by the housing system. LC sows lost 2.34 mm during farrowing, and CC sows lost 2.16 mm. The backfat loss depends mainly on body conditions at the beginning of farrowing, and the data obtained were similar for the sows involved in the trial, since 1 mm back fat is 7–8 kg body weight [41]; LC sows lost around 17.5 kg, and CC sows lost around 16.2 kg.

There were no differences between conventional and lift crate treatments for front/rear feet disease and locomotion scoring. This aspect is important in removing possible adverse effects of the lift crate on the crushing rate linked to sows’ feet diseases. In a recent study, front leg injuries were more critical than those detected on rear legs in crushing rates during farrowing, probably due to the difficulty of the sow to lift and turn to the rest position [12].

Only dew claw lesions seemed to rise, but not in a significant way, in LC crate sows, showing that the dynamics of the lift crate did not affect feet lesions or injuries. In general, both LC and CC crates resulted in a suitable housing system for feet conditions. However, it must be underlined that feet injury and lameness incidence rates usually depend on farm management for a wide variety of reasons [42,43]. Sows lodged in farrowing crates are not subjected to important risk factors for lameness [44,45], which usually occurs in the group housing system, mainly during aggressive attacks in order to establish a dominance hierarchy.

## 5. Conclusions

The lift crates showed remarkable efficiency in preventing piglet mortality by crushing, occurring mainly within 48 h after delivery, but the sows lodged in the lift crates showed an increase in hair cortisol concentrations during farrowing. These controversial results show that these confined farrowing systems can improve litter survival but with the effect of higher stress in sows, likely because of the dynamics of the lift farrowing cage. These results need to be deepened with further studies to investigate the reasons for the higher cortisol variations in lift crate-housed sows, comparing identically dimensioned crates, and considering other indicators to determine stress levels. 

## Figures and Tables

**Table 1:** Phases of the experimental study.

Monitoring Phase	Conventional Crate Rooms	Lift Crate Rooms
1	20 November–11 December 2018	13 November–4 December 2018
2	26 February–19 March 2019	19 February–12 March 2019
3	23 July–13 August 2019	16 July–6 August 2019

**Table 2:** Feet lesion scoring performed on sows upon entry and exit from the farrowing room.

Feet Lesion	Lesion Score According to Severity		
	Mild	Moderate	Severe
Toes (T)	One or more toes slightly longer than normal	One or more toes significantly longer than normal	Long toes that affect gait when walking
Dew Claws (DC)	Slightly longer than normal	Claws extend to floor surface when the pig is standing	Claw is torn and/or partially or completely missing
Heel Overgrowth and Erosion (HOE)	Slight overgrowth and/or erosion in soft heel tissue	Numerous cracks with obvious overgrowth and erosion	Large amount of erosion and overgrowth with cracks throughout
Heel-Sole Crack (HSC)	Slight separation at the juncture	Long separation at the juncture	Long and deep separation at the juncture
White Line (WL)	Shallow and/or short separation along white line	Long separation along white line	Long and severe separation along white line
Cracked Wall Horizontal (CWH)	Hemorrhage evident, short/shallow horizontal crack in toe wall	Long but shallow horizontal crack in toe wall	Multiple or deep horizontal crack(s) in toe wall
Cracked Wall Vertical (CWV)	Short/shallow vertical crack in wall	Long but shallow vertical crack in wall	Multiple or deep vertical crack(s) in wall

**Table 3:** Environmental conditions in the farrowing rooms.

Monitoring Phase	Mean Temperature, °C (Min–Max)		Mean Ventilation Rate, m^3^ h^−1^		Mean Relative Humidity, % (Min–Max)	
	Lift Crates	Conventional Crates	Lift Crates	Conventional Crates	Lift Crates	Conventional Crates
1	22.93 (20.80–29.20)	23.87 (21.30–28.90)	41.38 (29–72)	66.21 (27–77)	47 (33–66)	48 (35–64)
2	23.99 (21–28.7)	23.74 (24.4–29.3)	52.35 (18–59)	59.37 (22–63)	48.71 (34.1–63.7)	51 (37–68)
3	26.19 (25.00; 29.17)	26.44 (22.60–29.60)	303.00 (109–362)	322.27 (132–408)	48.51 (45–73)	51 (42–68)

**Table 4:** Crushed piglets during farrowing. All the litters were cross-fostered within 24 h after parturition; (a, b) data in the same column differ for *p* < 0.05.

	Born Alive Piglets/Litter	Born Dead Piglets/Litter	Mummified Piglets/Litter	Crushed Piglets/Litter within 72 h	Crushed Piglets From the 3rd Day to Weaning	Total Crushed Piglets/Sow	Weaned Piglets/Sow after Cross-Fostering
Lift Crates	14.67 (4.29)	1.39 (1.90)	1.24 (2.33)	0.15 (0.35) a	0.22 (0.81)	0.37 a (0.86)	12.27 (0.82)
Conventional Crates	14.55 (4.70)	0.76 (1.00)	0.39 (0.81)	0.44 (0.65) b	0.04 (0.13)	0.48 b (0.66)	11.87 (2.89)

**Table 5:** Mean hair cortisol upon entry to and exit from (and SEM) the farrowing room, and its variation in time; (a, b) data in the same column differ for *p* < 0.05.

Farrowing Crate	Mean HCC at the Entry in the Farrowing Room (pg/mL)	Mean HCC at the Exit from the Farrowing Room (pg/mL)	Mean Difference of HCC (Exit–Entry) (pg/mL)
Lift Crates	1.17 (0.12)	1.74 (0.16)	0.53 a (0.19)
Conventional Crates	1.37 (0.11)	1.60 (0.19)	0.22 b (0.19)

**Table 6:** Backfat thickness values measured in the back area, at point P2 of the rib, 6–7 cm lateral to the dorsal midline.

Farrowing Room	Mean of Backfat of Sows (mm, Entry)	Mean of Backfat of Sows (mm, Exit)	Mean of Backfat of Sows (mm, Variation)
Lift Crates	14.01 (0.88)	11.67 (0.65)	2.34 (0.75)
Conventional Crates	12.55 (1.61)	10.39 (0.68)	2.16 (1.03)

**Table 7:** Mean values of locomotion and feet disease scoring.

Crate	Time	Mean Sum of Toes	Mean Sum of Dew Claws (DC)	Mean sum of Heal Overgrowth and Erosion (HOE)	Mean sum of White Line (WL)	Mean sum of Hell-Sole Crack (HSC	Mean Sum of Cracked Wall Horizontal (CWH)	Mean Sum of Cracked Wall Vertical (CWV)
Lift Crates	in	1.32 (0.26)	1.64 (0.29)	3.68 (0.33)	1.24 (0.30)	3.32 (0.32)	0.84 (0.21)	0.72 (0.20)
	out	1.40 (0.26)	1.72 (0.29)	3.64 (0.33)	1.28 (0.30)	3.36 (0.32)	0.92 (0.21)	0.72 (0.20)
	variation	0.08	0.08	0.04	0.04	0.04	0.08	0
Conventional Crates	in	0.66 (0.26)	1.87 (0.30)	3.25 (0.34)	1.33 (0.31)	2.29 (0.33)	0.45 (0.21)	0.08 (0.20)
	out	0.62 (0.26)	1.87 (0.30)	3.37 (0.34)	1.33 (0.31)	2.29 (0.33)	0.50 (0.21)	0.08 (0.20)
	variation	0.04	0	0.12	0	0		0

## Data Availability

The data generated and analyzed during this study are included in this article.
